# Development of THz wave oscillation and its application to molecular sciences

**Published:** 2004-02-01

**Authors:** Jun-ichi Nishizawa

**Affiliations:** *) Semiconductor Research Institute, Kawauchi, Aoba-ku, Sendai, Miyagi 980-0862, Japan; **) Photodynamics Research Center, Sendai Branch of RIKEN, 519-1399, Aoba, Aramaki, Aoba-ku, Sendai, Miyagi 980-0845, Japan

**Keywords:** Terahertz wave, Raman laser, glass fiber, GaAs, GaP, LiNbO_3_, resonance spectroscopy, polymer, glucose, imaging, cancer

## Abstract

In 1967, a THz wave was generated for the first time using a free electron laser; however, the device was too expensive to be used widely. The author published the idea of THz wave generation by use of resonating vibration between paired atoms in polymer or crystal in 1965, and succeeded with K. Suto to generate a 12 THz wave in 1983. In 1999, the author invited K. Kawase and H. Ito to Sendai RIKEN to realize the concept by use of dielectrics; in 2000, they succeeded in realizing a sweep generator. In the same year, the author suggested the idea of applying the THz wave to cancer diagnosis and treatment, particularly by improving heating selectivity: This enhances the effect of medications by raising only the temperature of the cancer itself, not of the surrounding atomic pairs in the neighborhood. These applications are expected to spread quickly as powerful methodology based on molecular science. The reason for this is that the much improved waveform generated enables higher selectivity, allowing the detection of the existence of abnormal polymer near the paired atoms by measuring resonating frequency between paired atoms.

## Introduction

Influenced by conventional trends, researchers in electronics at Tohoku University were inclined to be interested in generating higher frequencies; following the work by Prof. K. Okabe, they had invented an anode split type magnetron, which is still in very wide use in microwave ovens. Prof. Okabe established a world record in generating highest frequency, which lasted for more than 20 years based on his invention of a series of new vacuum tubes, starting from 1927.

In 1957, the author received a hint for creating a semiconductor laser from a paper published by Wittke1) in *IRE Proceedings*; it concerned the realization of a semiconductor laser diode by the feeding of electrons in conduction band and holes in filled band, and the recombining of both as a result of induced recombination radiating stimulated emissions, a theory of which had already published by A. Einstein and printed in such book as *The Principles of Statistical Mechanics* by Tolman.2)

However, in those days in Japan, the author could not secure any financial support to perform the experiment. Then after 5 years, five research groups published their successes in realizing semiconductor lasers. These included IBM,3) Lincoln Laboratories in MIT,4) RCA,5) and General Electric,6) and were all the result of military contracts. The author was very disappointed. However, the editor, Yasuo Hirano, of a Japanese magazine *Electronic Science* asked the author to write an article explaining semiconductor lasers including future projects to be pursued.7)

The four project about which the author wrote were (1) optical glass fiber for optical communication, (2) nearly defect-free materials as GaAs8) for reliable devices for optical communication, and (3) development of terahertz (THz) wave generation which appeared possible by the use of peculiar resonance frequencies between atoms in a crystal or molecule. As the fourth project is yet to be undertaken, I will not comment on it here.

Given these orientations, the author attempted to develop glass fiber, for which he took out fundamental patents. It employed a concept of not only multimode but also such single-mode propagation as guided wave propagation. However, the author was not able to experimentally develop the fiber. The work ended on a theoretical note with the author having S. Kawakami write an analytical paper,9) which was two-dimensional and analogous with an analysis by Dr. Eaglesfield10) of gas lens.[Table tI-pjab-80-074]

Regarding the second project, fortunately my group, succeeded based on my concept in developing very high quality GaAs by applying optimum arsenic vapor pressure to maintain the equilibrium between the solid and gas phases. Accordingly, the highest quality GaAs crystals are being produced by Sumitomo, Japan, which are used in the manufacture of ultra-bright light emitting diodes and laser diodes.11)

## Development of THz oscillators

Our next project has been ongoing for about 40 years. It was about 20 years ago when Prof. K. Suto realized a Raman effect laser; mixing back this frequency with the original he was successful in inducing a 12 THz wave following the author’s guidance. The existence of a Stokes Raman Laser effect appeared to induce 12 THz lattice vibrations converted into an electric field.

In 2000, the author announced the application of THz waves in the detection of cancer, made possible by the result of measuring the spectrum induced by the resonance between two atoms. That is, every atom in a polymer or crystal always couples with neighbor atoms simultaneously. Therefore, single atoms should never be considered to couple with only one specific atom but rather with other atoms directly or indirectly. As this relation can be represented by a matrix, matrix dynamics are expected to be a very useful method of advancing research in this field.

The results should show the force of mechanical coupling between every two atoms. Therefore, it should be possible to estimate the mechanical field in the neighborhood of vacancies or of interstitials, not only the usual substitution impurity atoms, by estimating the resonance frequencies and their intensities. This should be an effective tool in the research of condensed state physics, liquid and solid. Figures 4 and 5 show some of the main resonating peaks, which are understood to be induced by the resonance between two atoms under normal circumstances. They appear to be followed by other peaks separated by energy induced by some defect atoms that differ from those under normal circumstances. Their height in the spectrum corresponds to the concentration of resonance induced by specific defects.

For these purposes, free electron lasers would seem to be more useful, as their measured values are more exact. However, our simple device, which is very small and easy to set up, makes it easy to measure the properties of the specimen. A free electron laser was first developed in roughly 1930; and in 1976, experiments by J. Madey at Stanford University generated about 30 THz oscillator using linear of 24 MeV. Of course, this equipment was very larger and heavy.

In 1998, K. Kawase and H. Ito were asked by the author to join the Photodynamics Research Center, the research direction of which had also been established by the author, where they performed the same THz oscillator by applying atomic vibration resonance oscillation using dielectrics to fill up the gap between higher electromagnetic wave frequency and laser light. In so doing, they succeeded in realizing a sweep oscillator using LiNbO_3_. Dr. Kawase then succeeded in improving the performance of the oscillator by introducing semiconductor laser light beam injection, allowing its output wave to be changed between 0.6–2.6 THz12) by adjusting the seeder wavelength or changing the angle. By the way, the corresponding value in the case of GaP Raman effect is about 0.3–7 THz, achieved by changing the angle or the power of the optical laser.13)

Upon the author’s advice, Dr. K. Kawase measured the absorption spectrum of gas phase H_2_O and HCl, which produced a very beautiful spectrum. The accuracy of the device’s resolution was about 100 MHz, which corresponds to the value of Q as 10^5^.14)

## Recent results of oscillations

**Fig. 1:**
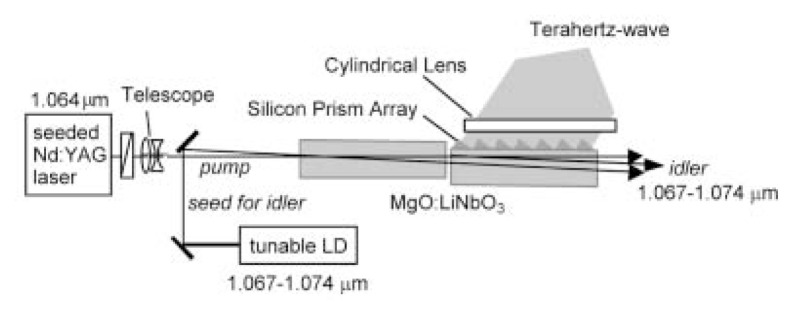
Injection-Seeded THz-wave Parametric Generator (IS-TPG).

**Fig. 2:**
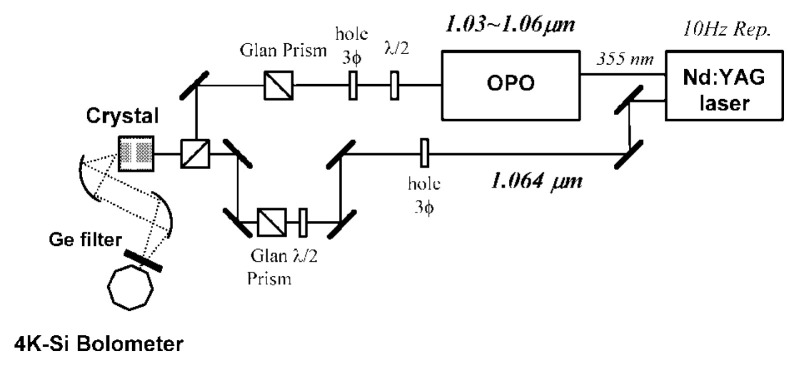
Schematics of the experimental set-up used for THz-wave generation in GaP crystals.

**Fig. 3:**
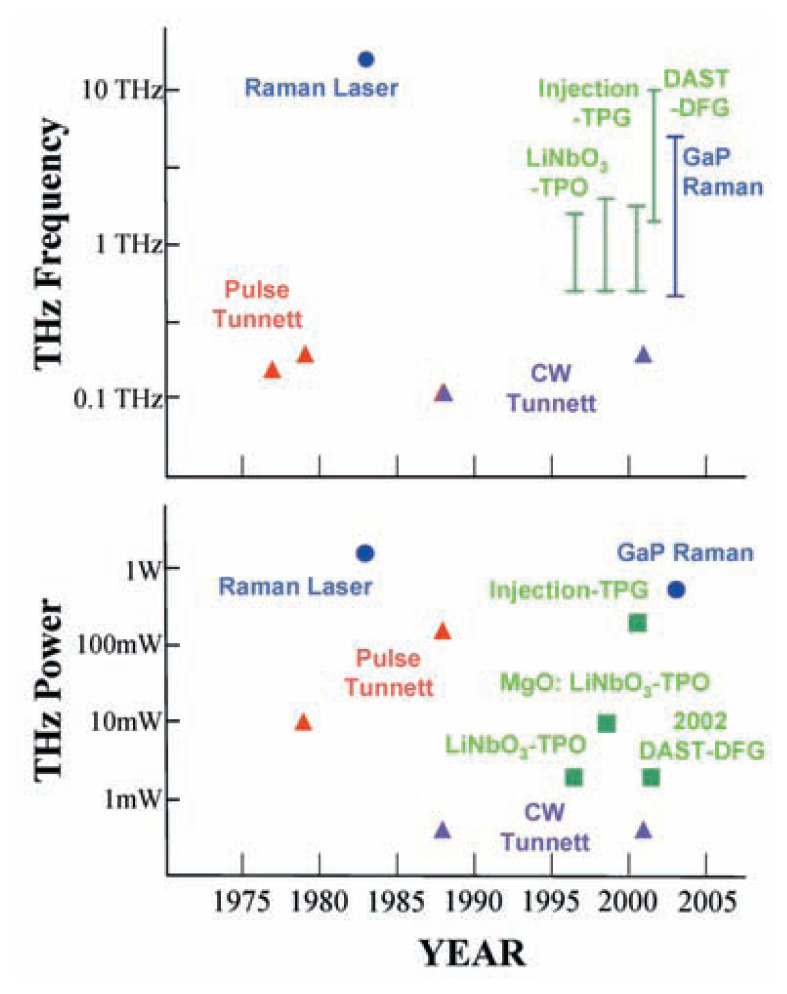
Progress of out put power and oscillating frequency per year.

A cooperative group directed by Prof. H. Nakanishi is eagerly preparing DASH (4-dimethylamino-N-methyl-4-stilbazalium tosylate) crystals, which are expected to be able to generate THz waves from 0.1 THz toward 10 THz. Already GaSe Raman effect generated THz sweep oscillation wave from 0.3 to 4.9 THz15) and other materials have been achieved. Others such as ZnS and ZnGeP_2_ are considered possible. Some of these have more than a little Antistokes and Stokes-coefficient in Raman effect. Usually, the resonance frequency of a solid can be evaluated by (k/M)^1/2^, where k is a spring constant and M is the mass of a solid atom coupled by spring constant k with the next atom. As an example, a microwave oven utilizes 2.45 GHz to induce resonance between one hydrogen atom and an oxygen atom. However, an atom in polymer or crystal cannot be considered so simply as it is connected by several springs with neighbor atoms and again with next neighbor atoms. These springs represent a balance between the van der Waals force and binding force. Whereas the total parallel springs strengthens the overall spring constant, total effective mass is very difficult to understand. It is difficult to explain why it appears to be small and why its resonance frequency becomes high. However, resonance frequencies in a polymer or solid are very difficult to perceive. Even in a large polymer, resonance frequency is usually only as high as THz. In the future, we should work eagerly to advance our analysis of these mechanical properties by using matrix dynamics. Recently, our analysis of protein using resonance spectroscopy seems to be progressing very successfully. This will be reported by Prof. H. Komatsu16) of my group. Figures 1, 2 and 3 show examples of the structure of our oscillator set. For these we heartily express our thanks to Japanese Ministry of Postal and Telecommunications, Japanese Ministry of Education, Science, Sports and Culture, and Japanese Science and Technology Agency.

## Results of measurement

THz waves can, for example, be applied to very thin film of polymer on a plastic plate, allowing their transmittance to be measured by a PbSnTe photo diode or silicon borometer.

**Fig. 4:**
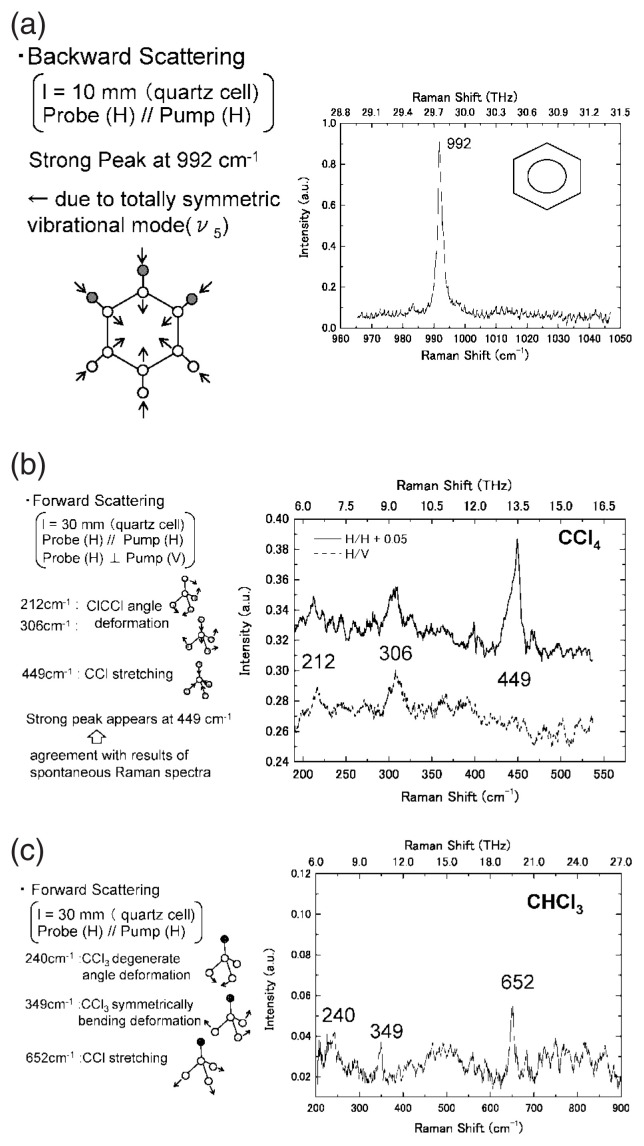
(a) Stimulated Raman spectrum of benzene. (b) Stimulated Raman spectrum of carbon tetrachloride. (c) Stimulated Raman spectrum of chloroform.

Figure 4(a) shows the result in the case of Benzene in quartz cell, where the peak appears to occur in a totally symmetric vibrational mode, which resonate at 992 cm^−1^. Figure 4(b) shows the result in the case of carbon quarto chloride, where the resonance at 212 cm^−1^ and 306 cm^−1^ appears to correspond to angle deformational vibrations, and the resonance at 449 cm^−1^ to the stretching vibration of 4 chlorine atoms toward the central carbon atom. Figure 4(c) shows the result in the case of chloroform, where the transmission peak at 240 cm^−1^ appears to corresponds to the degenerate angle deformational mode, 349 cm^−1^ to the symmetrically bending deformational vibration, and 652 cm^−1^ to the stretching vibration. Details will be published by T. Tanabe *et al.* of my group.

**Fig. 5:**
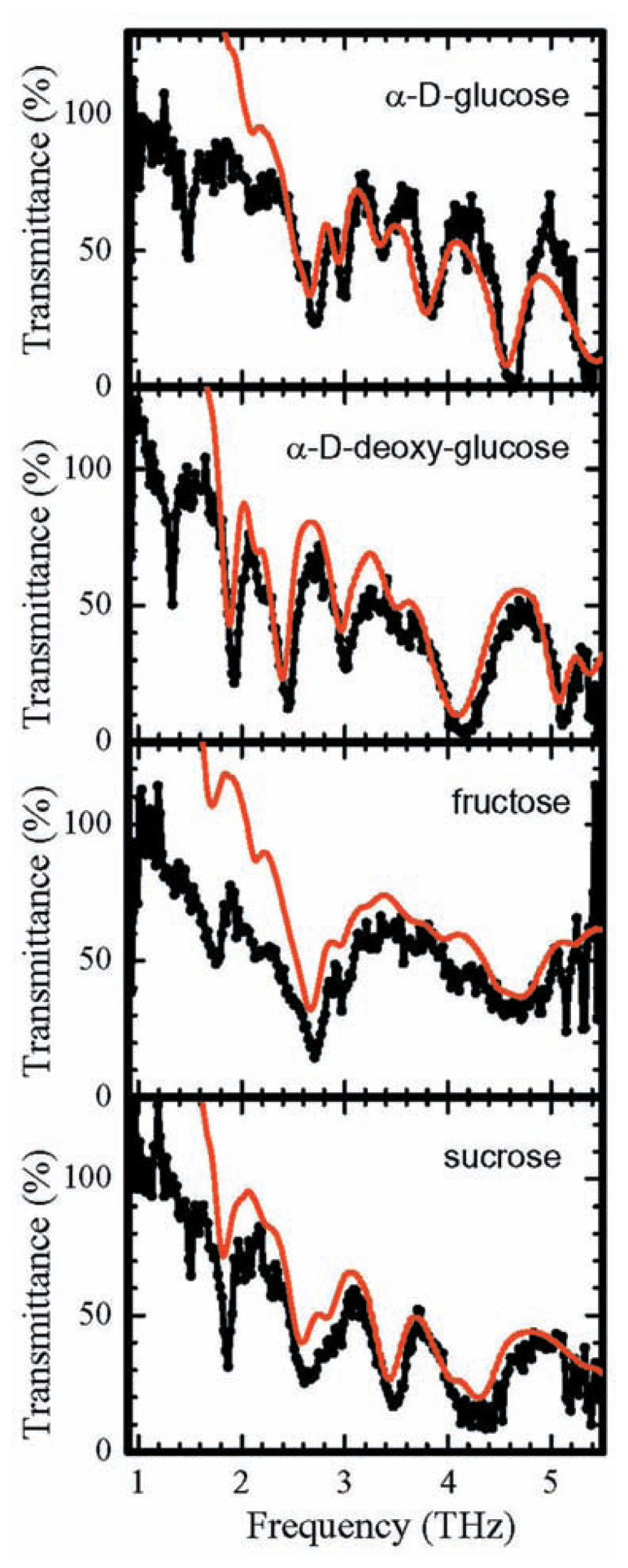
Transmittance of sugars in THz band. Black lines indicate spectra measured by GaP THz generator and red lines indicate spectra by FTIR.

Some other vibrations with distinctive bonds have already been introduced in a proceedings paper.17) Such data must be accumulated before conducting an analysis of polymers or crystals. However, Fig. 5 shows the results of our measurement using a GaP Raman oscillator and compares them with the results using a generator synthesized by mixing femto second pulses. The latter gives only very approximate, imprecise results, and moreover in many cases exceeds the value 100%, which is impossible. Therefore, the results are inaccurate, which is also the case with those of FTIR. On the other hand, the GaP Raman method yields very precise results, which allows analysis and each peak to be connected to a specific vibration. As already mentioned, even vacancies are expected to be detected. These results can determine very easily the kind of sugar; and, if a particular frequency is chosen, will in the future be able to make the determination in just a few seconds.

**Fig. 6:**
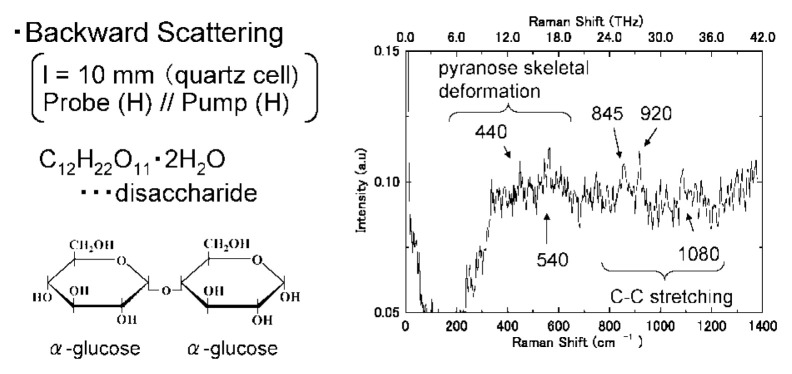
Stimulated Raman spectrum of trehalose in water.

Figure 6 shows the case of ***α*** glucose (Trehalose), where vibrations at 440 and 540 cm^−1^ appear to be caused by skeletal deformation of the piranose ring structure, with those at 845, 920, and 1080 cm^−1^ appearing to correspond to the stretching vibration of carbon to carbon bonding. Details will be published by T. Tanabe *et al.* of my group.

**Fig. 7:**
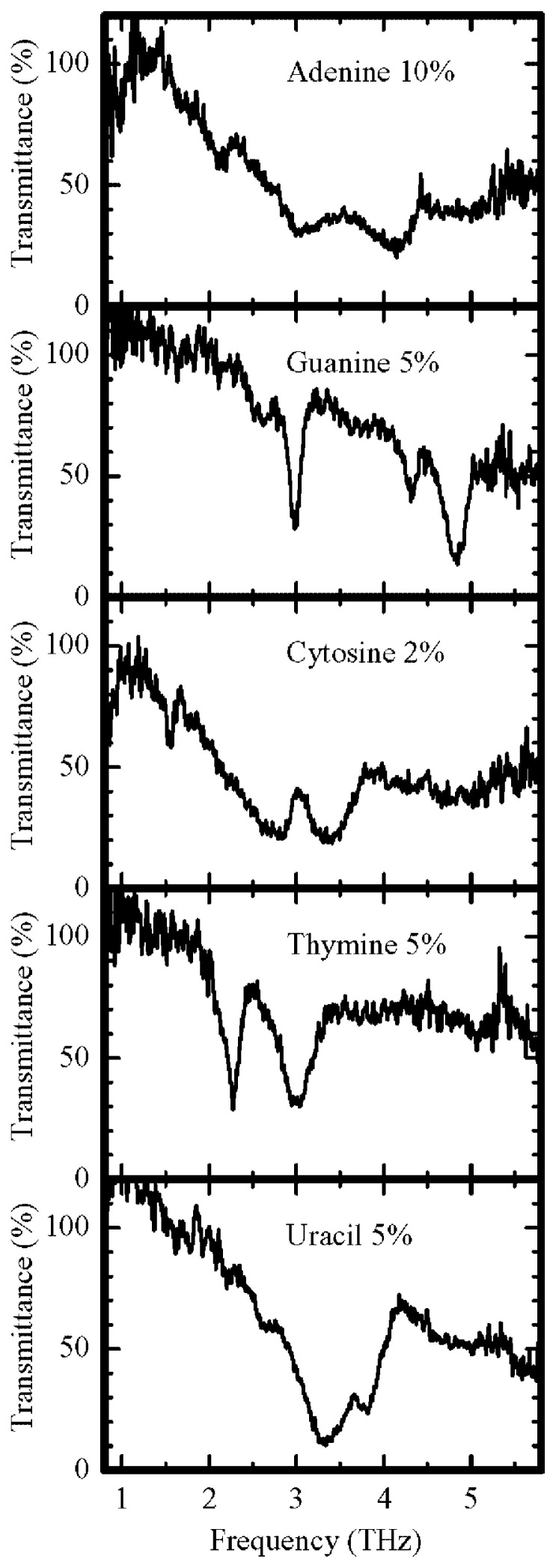
Transmittance of nucleo bases. Adenine, Guanine, Cytosine, Thymine and Uracil are components of DNA and RNA molecules.

Furthermore, the results of similar experiments on important materials used in biochemistry are shown in Fig. 7. They could be measured without any damage or defect being caused by the measurement. These data and their analyses should be of increasing importance, particularly because their characteristics change in interactions with other biomolecules, bacteria or viruses. Details will be published by T. Sasaki *et al.* of my group.

**Fig. 8:**
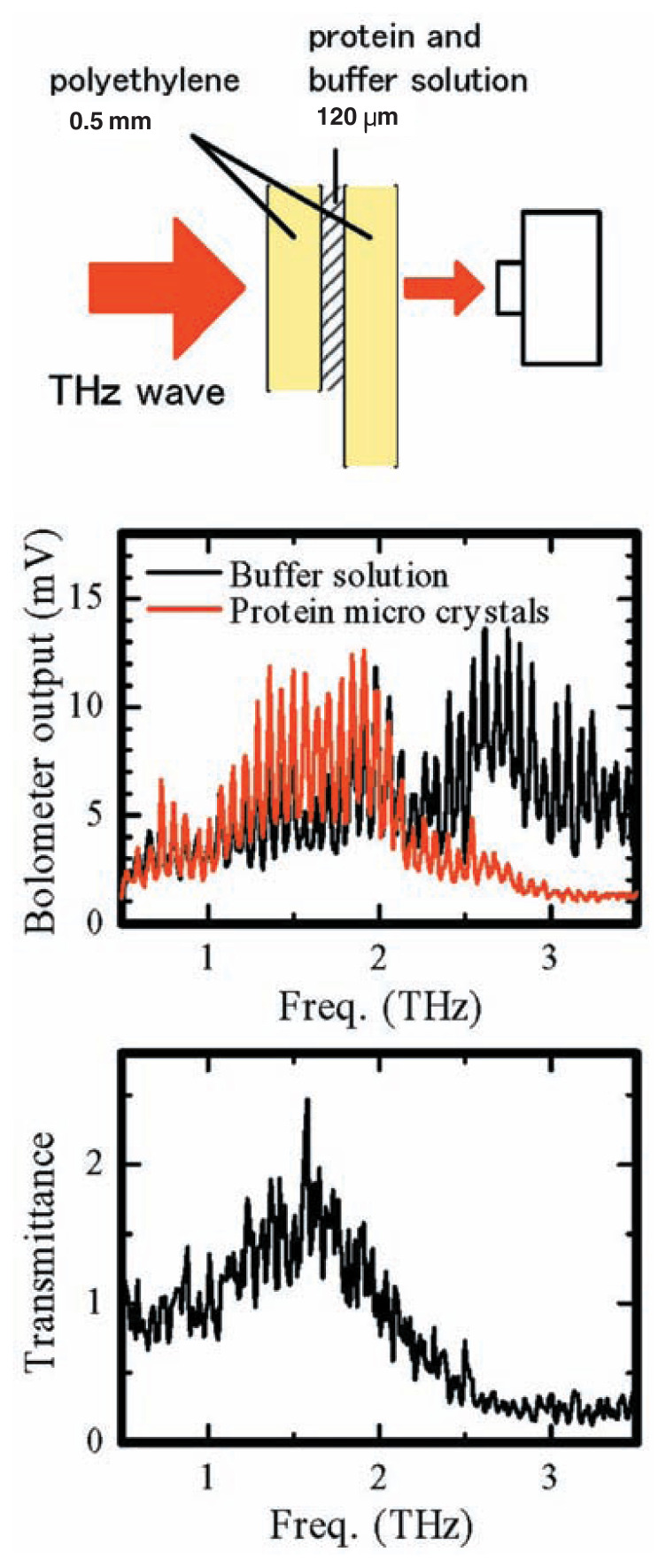
Transmittance of protein micro crystals. THz wave passed through solution of its thickness over 100 μm could be detected.

It might be surprising that such a simple and portable device can observe such detailed precise data, enabling even more precise analysis of polymers and organic chemical compounds in the future. Figure 8 shows the measurement results of the transmission coefficient of protein. Very rapidly, these measurements are likely to be applied in a wide area of fields. By expanding that area by accumulating experimental results over a wide spectrum of fields and, as already described, analyzing the meaning of each measured result, it will be possible to take large-scale and very effective measurements.

## Imaging

Imaging technology has been rapidly developed based on CCD technology. Unfortunately, however, photo diode materials sensitive to THz waves are usually of a very narrow bandwidth, which makes it difficult to fabricate a device made of narrow bandwidth material that can produce THz images and covert THz wave detections and images toward time series pulse trains as with CCD. In my laboratory, we are working with simple methods to develop such a device, but have yet to finish it. Details will be published by T. Sasaki *et al.* of my group.

**Fig. 9:**
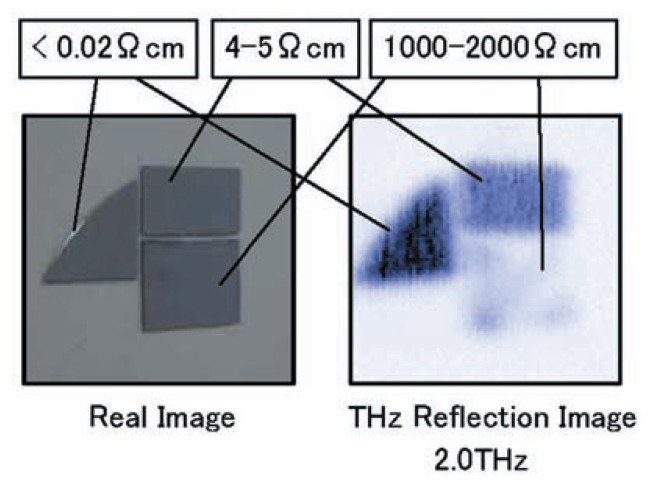
THz reflection images of Si wafers. Thickness of color shows THz wave intensity. The wafer with low resistivity showed high reflectivity.

**Fig. 10:**
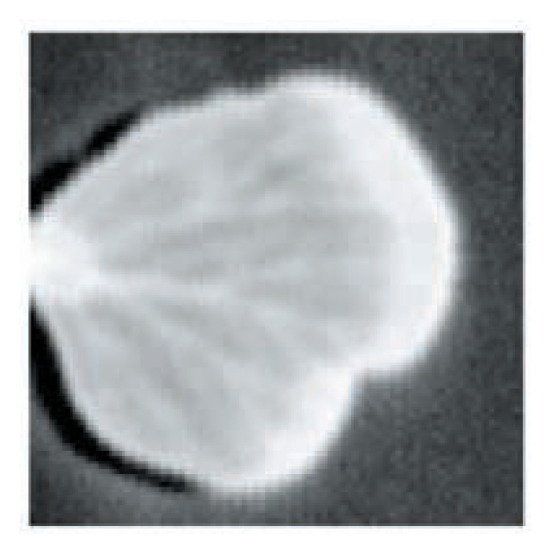
THz transmission image of a leaf. The veins of the leaf could be observed.

**Fig. 11:**
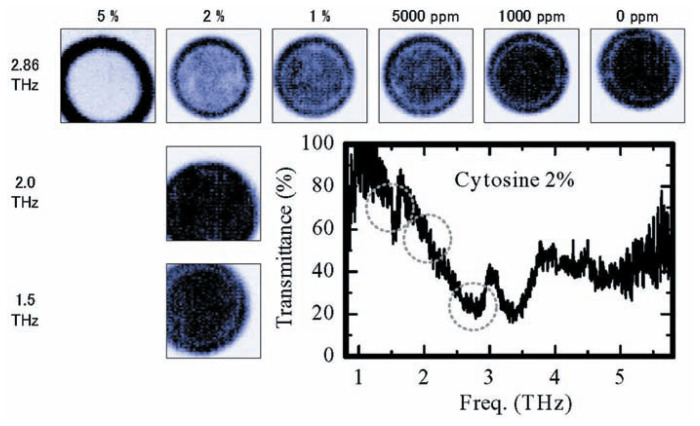
THz transmission images of polyethylene pellets containing cytosine. The pellets with its contains of Cytosine under 1% be detected.

Figure 9 shows the imaging of silicon wafers and reveals the distribution of silicon resistively. Figure 10 shows the transmitted imaging of plant leaf, while Fig. 11 reveals the pattern of distributed cytosine, which with the same time sensitivity can be measured in an amount of approximately 1000 ppm. In the future, however, it will be necessary to more precisely select the peak in the measurement frequency, which will necessitate setting standards for each peak. In other words, today’s measurements are only approximately qualitative. Details will be published by T. Sasaki *et al.* of my group.

**Fig. 12:**
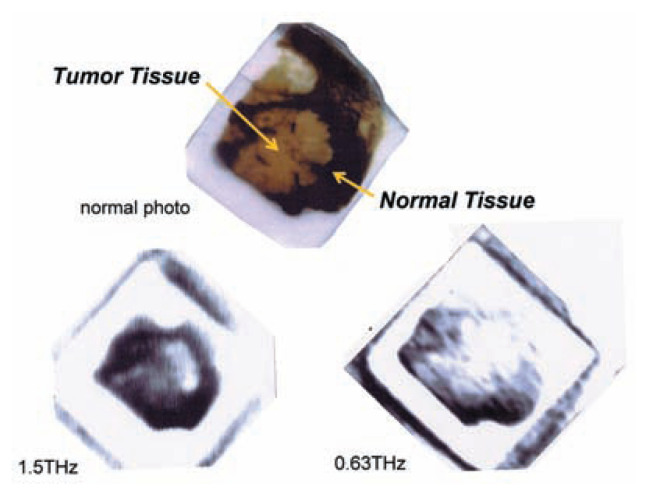
Partly cancered liver changes the reflection with higher frequency.

Finally, measurement of the cancerous part in the liver is of great importance; therefore, the author again refers to the picture in Fig. 12. The most important results are the changing properties of the cancerous part and the non-cancerous part. The not-yet cancerous part changes transmittance with the chances in frequency, but not the cancerous part. Of course, spectrum in a very important tool today for determining whether or not cancer exists. In the future, however, optimum selection of the frequency will make it possible to simplify the device. By applying very strong irradiation at a specific frequency, it will be possible to destroy the cancerous area without destroying the non-cancerous area, due to the effect of highly localized heating through induced vibration at the cancerous part. In the case of medication as well, injections can be given with reduced side effects.

## Molecular science

Over many years, consideration has often been given merely to the molecule. In the future, the molecule must be the target of much deeper analysis. As was already shown, sugar was identified and then in a nondestructive examination, the vibration of one paired atoms in a polymer or crystal was found to be influenced by each neighbor atom, allowing the point defect in the material to be reversely examined.

That is, an examination can even be made of the molecular structure. As an example, by conducting a spectrum comparison of the SARS virus with the normal pneumonia virus, differences in their molecular structure can be ascertained. The same applies to other diseases such as BSE. This makes it possible to estimate the molecular structure of the bacterium or virus and, in turn, the molecular structure of corresponding medications.

Furthermore, it will be possible in the same way to determine the structure of cancerous molecules. Then, if a wave of the appropriate frequency is applied to the cancerous molecules, only those molecules will be heated, without damaging other healthy molecules.

If we place the device at the entrance of a vegetable market, O157 and other types of bacteria can be detected very easily, as can be anthrax at post offices and other places.

For these purposes, reflection-type measurement devices would be easier to apply than transmission-type devices. Figs. 8–12 shows the results of a preliminary experiment on measurement devices.

## Future

In the future, I expect a very quick spread of academic interest in this field, not only in biophysics, but spanning agriculture, medication, diagnosis, treatment, bacteriology, physiology, pathology and biochemistry. It will generate a much deeper and wider understanding especially of biosciences.

## Figures and Tables

**Table I tI-pjab-80-074:** Chronological table for terahertz generation

1910	D. Hondros & P. Debye	Dielectric waveguide
1936	T. Seki & H. Seimiya	Light transmission via dielectric rod
1954	M. T. Weiss & E. M. Gyorgy	Dielectric waveguide
1957	J. Nishizawa	Proposal of laser, in particular, semiconductor laser
1958	A. L. Shawlow & C. H. Townes	Proposal of gas laser
1960	T. H. Maiman	Realization of ruby laser
1961	A. Javan	Realization of gas laser
1962	M. I. Naithan, R. N. Hall, T. M. Quist, *et al*.	Realization of semiconductor laser
1963 April	J. Nishizawa	Proposal of terahertz wave generation via molecular and lattice vibrations (*Denshikagaku* p. 17, p. 30)
1963 May	R. Loudon	Proposal of terahertz wave generation via molecular and lattice vibrations
1964	J. Nishizawa & I. Sasaki	Proposal of optical fiber communication and focusing optical fiber (Diminution of Leakage Loss)
1965	J. Nishizawa	Proposal of terahertz wave generation via molecular and lattice vibrations together with tunneling (*Denshigijutsu*)
1966	K. C. Kao	Estimation of low absorption loss optical fiber
1969	R. H. Pantell	Observation of frequency shift via lattice vibrations
1971	J. Nishizawa	Proposal of generation of terahertz wave as a result of mixing of original wave with the wave induced by Raman effect
1973	P. P. Solokin, J. J. Wynne & J. R. Lankard	Four wave parametric effect in alkaline metals
1973	J. Nishizawa	Proposal of Ideal Static Induction Transistor (Ballistic SIT) as terahertz operating devices
1979	J. Nishizawa & K. Suto	Realization of semiconductor Raman laser with lattice vibration
1979	J. Nishizawa	Realization of TUNNETT diode oscillating at 0.33 THz
1983	K. Suto & J. Nishizawa	Difference frequency wave generation via semiconductor Raman laser
1997	K. Suto & J. Nishizawa	Semiconductor waveguide Raman amplifier
1999	J. Nishizawa, P. Plotka & T. Kurabayashi	Realization of Ballistic SIT (scattering free SIT)
2000	K. Kawase & H. Ito	THz wave parametric generation by injection seeding
2000	J. Nishizawa	Proposal of application of THz wave to diagnosis and medical treatment of cancer
